# Influence of different incision designs on bone increment of guided bone regeneration (Bio-Gide collagen membrane +Bio-OSS bone powder) during the same period of maxillary anterior tooth implantation

**DOI:** 10.1080/21655979.2021.1932209

**Published:** 2021-05-30

**Authors:** Genying Zhuang, Jianshui Mao, Guoli Yang, Huiming Wang

**Affiliations:** a Department of Stomatology, The Fourth Affiliated Hospital of Zhejiang University School of Medicine, School of Stomatology, Yiwu, China; b Key Laboratory of Oral Biomedical Research of Zhejiang Province, Hangzhou, China; c Department of Orthopaedics, The Fourth Affiliated Hospital of Zhejiang University School of Medicine, Yiwu, China; d The Affiliated Hospital of Stomatology, School of Stomatology, Zhejiang University School of Medicine, Hangzhou, China

**Keywords:** Incision design, bone transplantation, maxillary anterior teeth, dental implants

## Abstract

Exploring the influence of different incision designs on bone increment of guided bone regeneration [Bio-Gide collagen membrane +Bio-OSS bone powder (carbonate apatite crystal extracted from bovine bones), Bio-OSS bone meal was placed on the surface of the bone defect and then covered with a Bio-Gide membrane to close the wound] during the same period of maxillary anterior tooth implantation. The 99 patients from the stomatology department were divided into 3 groups: small incision (N = 30, group A), wide incision (N = 39, group B), internal gingival sulcus incision (N = 30, group C). At the different time (immediately after surgery, 6 months, 12 months and 24 months), the width and height of labial bone at different implant margin (2 mm, 4 mm, 6 mm) has no significant difference in comparison of any two of the three groups (p > 0.05). The score of esthetic feeling in group A was significant higher than group C (P < 0.05). The PPD, the incidence of SH, BOP in group A were all significant higher than group B (P < 0.05). The PISm, PISd, PPD, the incidence of SH and BOP in group A were all significant higher than group C (P < 0.05). The PISm, PISd, PPD, the incidence of SH and BOP in group B were all significant higher than group C (P < 0.05). The three groups has no significant different on the influence bone increment. The soft tissue condition around the implant after surgery was better in internal gingival crevicular incision than others two incisions, large-scale incision better than small incisions.

## GRAPICAL ABSTRACT

**Figure:**
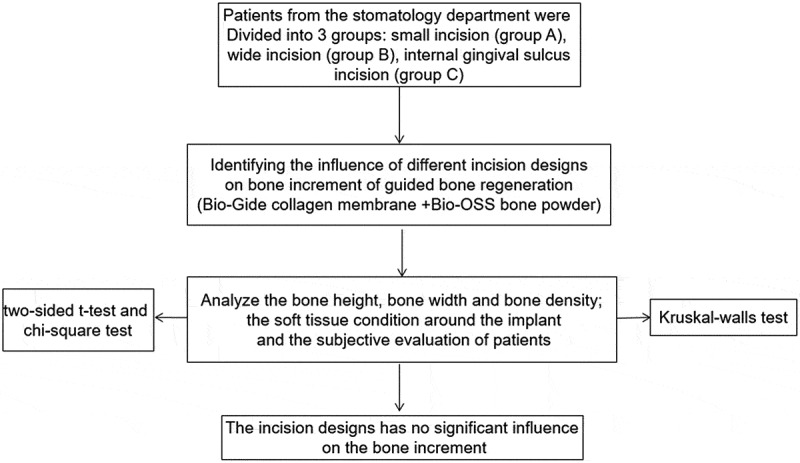

## Introduction

After maxillary tooth loss, the masticatory function and quality of life of patients will be seriously affected. If partial removable denture is used for repair, satisfactory results are difficult to be achieved, while fixed bridge repair requires high requirements on the surrounding remaining teeth and is especially damaged. Therefore, implantable denture has gradually become the main way of repairing dentition defect and dentition loss, especially for patients with terminal-free loss. In the area of after maxillary teeth, special anatomic factors, maxillary sinus gasification and tooth extraction could lead to the severe alveolar bone absorption (mostly because of severe periodontal inflammation). Improper removal of partial denture, hormone metabolism and others reasons would caused bone absorption and bone atrophy. Osteoporosis in the base area of the maxillary sinus would significantly increased the difficulty of dental implants [1–4].

With the rapid development of bone-binding technology, dental implant has become one of the conventional methods for repairing dentition defects and missing teeth. However, the lack of alveolar ridge width, low level of maxillary sinus floor and mandibular defects are common in clinical practice, resulting in insufficient bone mass around the implant. Therefore, many bone graft materials and techniques have been used to increase the height and width of alveolar bone. Deproteinized natural bovine mineral (Bio-OSS) bone powder is a carbonate apatite crystal extracted from bovine bone [5–8]. After special treatment, protein and other organic components are removed, which is almost the same with the structure of human bone. At present, Bio-OSS bone powder is the most widely used bone graft material in the dental implantation process. Bio-OSS bone meal is one of the most widely used artificial bone materials in GBR. It is derived from natural bovine bone, and its inorganic structure is almost similar to the inorganic structure of human bone (low-crystal natural phosphorite). The structure and large surface area provide an ideal frame structure for the regeneration of new bone. Bio-OSS bone meal has excellent biocompatibility due to the complete removal of organic matter [9–11].

In this study, we aimed to evaluate the influence of different incision designs (small incision, wide incision and internal gingival sulcus incision) on bone increment of guided bone regeneration (bioresorbable collagen membrane (Bio-Gide) collagen membrane +Bio-OSS bone powder) during the same period of maxillary anterior tooth implantation. We assumed that the internal gingival sulcus incision group had a higher postoperative bone gain than the other two groups, and the complication rate in the wide incision group is higher than that in the other two groups.

## Materials and methods

### Study population

This retrospective cohort study was conducted from May 2013 to December 2019 at the Department of stomatology. The study was approved and monitored by the Medical Ethics Committee of Stomatological Hospital Affiliated to Zhejiang University School of Medicine (Approval No. 26 in 2020). Patients, who were admitted to the stomatology department, were recruited if they met the following criteria: (1) Age between 18 and 65, regardless of gender. (2) The defect of maxillary anterior denture should be repaired by implant fixation. (3) Patients who meet the indications for implantation, have no systemic diseases and can withstand implantation surgery. (4) Periodontal condition was good, without progressive alveolar bone resorption or periodontal abscess. (5) Patients with horizontal bone defects (referring to any specific part, e.g. alveolar bone resorption) in the implant area who need guided bone regeneration. (6) There is at least one bone defect but no more than three bone defects in the remaining alveolar bone wall in the tooth missing area, and there is no adequate bone inclusion around the implant. (7) No looseness of adjacent teeth in the missing tooth area. (8) Patients with good oral environment and good compliance. (9) Sign the informed consent of surgery voluntarily.

Exclusion criteria included the following: (1) The remaining four bone walls at the tooth extraction fossa site were not required to guide bone regeneration. (2) Metal PFM crown or metal casting pile is used for the adjacent teeth in the missing area. (3) Patients with serious periodontal disease and unstable periodontal condition. (4) patients with acute or severe infection and white blood cell count of >10 × 10^9^/L. (5) Pregnant and lactating women. (6) Long-term use of special drugs such as hormones, anticoagulants, bisphosphonates, etc. (7) Patients with poor systemic and nutritional status and cannot tolerate dental implant operation. (8) Patients who have a history of heart disease or arteriosclerosis and cannot tolerate dental implant operation. (9) Patients with blood system diseases and uncontrolled cardiovascular diseases (hypertension [systolic blood pressure >150 mmHg, diastolic blood pressure b > 90 mmHg], coronary heart disease, congenital heart disease, etc.). (10) Systemic or local bone diseases are considered as contraindications for implant repair, such as bone tuberculosis, osteitis, and bone tumor. (11) persons suffering from systemic immune diseases. (12) persons with severe epilepsy or mental illness or mental disorder. (13) fasting blood glucose >7.0 mmol/L in patients with uncontrolled diabetes. (14) Patients with head and neck diseases with a history of radiotherapy within 5 years. (15) The blood coagulation time is more than twice the normal range. (16) The researcher considered it inappropriate to participate in this clinical study for other reasons.

### Grouping design

According to the incision designs, the 99 patients from the stomatology department were divided into 3 groups: small incision (medial gingival papilla incision, N = 30, group A), wide incision (angle or trapezoidal incision, N = 39, group B), internal gingival sulcus incision (N = 30, group C).

In group A, the horizontal incision at the crest of the alveolar crest was added to the proximal and distal vertical incision on the medial side of the gingival papilla, and the vertical incision extended to the joint of the gingival membrane and gingival at the lateral lip.

In group B, in addition to the horizontal incision at the top of the alveolar ridge in the operative area, the incision was extended to 1–2 tooth positions outside the operative area for unilateral or bilateral vertical incision, and the vertical incision was extended to the joint of the gingival membrane and gingival at the lip side.

In group C, according to the scope of the bone defect, the incision was designed to extend the horizontal incision of the alveolar crest in the operative area to the gingival crevicular two to three or even more teeth outside the bilateral operative area, without vertical incision.

### Treatment procedures

Routine disinfection towel, local infiltration anesthesia, groove incision, gingival separation, minimally invasive tooth extraction. The mucoperiosteum flap was opened through different incisions in the three groups. Scratch and scrape tooth extraction nests for thorough debridement; to determine the defect of bone; the alveolar bone wall was gently scraped so that blood oozed. The new blood was collected with a 1 mL syringe and mixed with Bio-OSS granules. The mixed Bio-OSS particles were implanted into the tooth extraction fossae to make the bone graft material level with the highest point of the bone crest. Bio-Gide collagen membrane should be trimmed to cover the surface of bone graft material, at least 2 ~ 3 mm beyond the edge of bone defect. Periosteum dilatation incision was made to relax the gingival flap, and the gingival flap was reduced to the crown square after close suture to completely close the wound [12–14].

### Radiographic measurements

On the day after the operation and 6 months, 12 months and 24 months after the operation, the following indexes were measured at fixed points after the registration of each CBCT tomography (near, central and far sagittal planes): the width of the labial bone at different implant margin (2 mm, 4 mm, 6 mm), the height of the labial bone at different implant margin (2 mm, 4 mm, 6 mm).

### The state of soft tissue surrounding the implant

The peri-implant soft tissue status of the three groups was observed from the Mesial papilla index (PISm), Distal papilla index (PISd), pocket probing depth (PPD), Scar hyperplasia (SH) and Bleeding of probing (BOP) respectively. The scoring criteria used Jemt (1996) the PIS: 0 point (no gum papillary); 1 point (the gingival nipple is no more than half the height of the adjacent space); 2 points (the gingival papilla is filled with more than half the height of the adjacent space, but does not reach the adjacent tooth contact); 3 points (the gum nipple is full of the entire adjacent teeth); 4 points (hyperplastic gingival papilla).

### Patient satisfaction

The patient satisfaction was measured according to the scoring method: satisfied 5 points, relatively satisfied 4 points, basically satisfied 3 points, relatively dissatisfied 2 points, and dissatisfied 1 point. The six dimensions of the implant function load, including esthetics, mastication, stability, ease of cleaning, comfort, language and food impaction, were measured respectively.

### Statistical analysis

Statistical analyses were completed using the SPSS 22.0 software. Data were reported as mean (standard deviation) or number (%). Outcomes were compared between two groups with two-sided t-test for continuous variables and chi-square test for categorical variables. Kruskal-Wallis test was used for comparison among the three groups. All tests were two sided and P < 0.05 was considered to be statistically significant.

## Results

We aimed to evaluate the influence of incision designs on bone increment of guided bone regeneration during the maxillary anterior tooth implantation. We assumed that the internal gingival sulcus incision group had a higher postoperative bone gain than the other two groups, and the complication rate in the wide incision group is higher than that in the other two groups. The specific test results are described in detail below.

A total of 99 patients (55 male, 44 female) were included in the present study. By the incision design, 30 patients in the group A (small incision), 39 patients in the group B (wide incision), 30 patients in the group C (internal gingival sulcus incision). The tooth loss and tooth position has also presented in Table 1. We assumed that the internal gingival sulcus incision group had a higher postoperative bone gain than the other two groups, and the complication rate in the wide incision group is higher than that in the other two groups.

**Table 1.** Basic information of patients

Index	A(n = 30)	B(n = 39)	C(n = 30)	*p*
Age(year)(mean±sd)	34.6 ± 11.5	37.1 ± 11.9	38.6 ± 11.5	0.423
Gender(M/F)	12/18	25/14	18/12	0.115
Smokers[n(%)]	6(20.0)	11(28.2)	11(36.7)	0.358
Tooth loss in the planting area[n(%)]				
Single tooth	25(83.3)	21(53.8)	14(46.7)	0.024
Continuous multiple teeth	2(6.7)	14(35.9)	12(40.0)	
Indirect multiple teeth	3(10.0)	4(10.3)	4(13.3)	
Tooth position[n(%)]				
11	10(33.3)	11(28.2)	2(6.7)	<0.001
12	0(0)	1(2.6)	4(13.3)	
13	3(10.0)	0(0)	0(0)	
21	12(40.0)	8(20.5)	7(23.3)	
22	0(0)	1(2.6)	1(3.3)	
11,21	2(6.7)	7(17.9)	12(40.0)	
11,21,22	0(0)	1(2.6)	0(0)	
11,22	0(0)	2(5.1)	0(0)	
12,11,21	0(0)	4(10.3)	0(0)	
12,21,22	0(0)	0(0)	4(13.3)	
13,21	3(10.0)	0(0)	0(0)	
21,22	0(0)	4(10.3)	0(0)	

## Width of the labial bone at different implant margin

The width of labial bone at different implant margin (2 mm, 4 mm, 6 mm) in different time (immediately after surgery, 6 months, 12 months and 24 months) has presented in Table 2. At the different time, the width of labial bone at different implant margin has no significant difference in comparison of any two of the three groups (p > 0.05).

**Table 2.** Comparison of the width (mm) of the labial bone measured by CBCT at different implant margin (2 mm, 4 mm, 6 mm)

Time	A(n = 30)	B(n = 39)	C(n = 30)	*p*		
				A vs B	A vs C	B vs C
Immediately after surgery						
IM2	3.06 ± 0.88	3.12 ± 0.93	3.11 ± 1.05	0.971	0.990	0.999
IM4	3.35 ± 0.80	3.76 ± 0.90	3.48 ± 1.07	0.248	0.930	0.677
IM6	3.28 ± 0.92	3.87 ± 0.98	3.55 ± 1.28	0.113	0.800	0.693
6 months						
IM2	2.28 ± 0.66	2.22 ± 0.77	2.34 ± 1.09	0.951	0.986	0.930
IM4	2.74 ± 0.84	2.80 ± 0.87	2.87 ± 1.23	0.966	0.948	0.985
IM6	2.64 ± 0.93	3.11 ± 0.88	3.00 ± 1.14	0.223	0.629	0.948
12 months						
IM2	2.02 ± 0.74	1.81 ± 0.74	2.12 ± 1.12	0.635	0.962	0.642
IM4	2.28 ± 1.00	2.28 ± 0.76	2.67 ± 1.20	0.999	0.634	0.534
IM6	2.40 ± 0.94	2.62 ± 0.87	2.70 ± 1.12	0.712	0.732	0.971
24 months						
IM2	1.73 ± 1.01	1.47 ± 0.81	1.92 ± 1.21	0.642	0.892	0.432
IM4	1.99 ± 1.11	2.00 ± 0.88	2.19 ± 1.24	0.975	0.655	0.609
IM6	2.18 ± 1.02	2.24 ± 0.93	2.45 ± 1.15	0.981	0.794	0.821

*IM2: 2 mm below implant margin; IM4: 4 mm below implant margin; IM6: 6 mm below implant margin.

The width change of labial bone at different implant margin (2 mm, 4 mm, 6 mm) in different time (immediately after surgery, 6 months, 12 months and 24 months) has presented in Table 3. From the Table 3, we found that the width change of labial bone has no significant difference in comparison of any two of the three groups (p > 0.05).

**Table 3.** Comparison of the width (mm) change of the labial bone measured by CBCT at different implant margins (2 mm, 4 mm, 6 mm)

Time	A(n = 30)	B(n = 39)	C(n = 30)	*p*		
				A vs B	A vs C	B vs C
6 months						
IM2	−0.78 ± 0.54	−0.91 ± 0.70	−0.77 ± 0.56	0.762	0.999	0.768
IM4	−0.61 ± 0.47	−0.96 ± 0.77	−0.62 ± 0.71	0.124	0.999	0.322
IM6	−0.64 ± 0.80	−0.76 ± 0.67	−0.55 ± 0.61	0.874	0.931	0.548
12 months						
IM2	−1.05 ± 0.73	−1.31 ± 0.70	−1.00 ± 0.57	0.449	0.980	0.255
IM4	−1.07 ± 0.70	−1.48 ± 0.85	−0.82 ± 0.89	0.180	0.686	0.069
IM6	−0.88 ± 0.81	−1.25 ± 0.81	−0.85 ± 0.76	0.302	0.996	0.263
24 months						
IM2	−1.33 ± 0.97	−1.67 ± 0.80	−1.19 ± 0.67	0.489	0.888	0.115
IM4	−1.36 ± 0.83	−1.76 ± 0.97	−1.29 ± 0.90	0.131	0.832	0.110
IM6	−1.10 ± 0.80	−1.64 ± 0.90	−1.10 ± 0.99	0.103	0.999	0.225

*IM2: 2 mm below implant margin; IM4: 4 mm below implant margin; IM6: 6 mm below implant margin.

## Height of the labial bone at different implant margin

The height change of labial bone has accessed at two aspects: the mesial aspect and the distal aspect. The height change of labial bone has presented in Table 4. The height change of labial bone at 6 months, 12 months and 24 months has no significant difference by pairwise comparison (P > 0.05).

**Table 4.** Comparison of the height (mm) change of the labial bone measured by CBCT at different implant margins (2 mm, 4 mm, 6 mm)

Time	A(n = 30)	B(n = 39)	C(n = 30)	*p*		
				A vs B	A vs C	B vs C
6 months						
MH plate C	0.03 ± 0.11	0.02 ± 0.13	0.03 ± 0.07	0.776	0.999	0.725
DH plate C	2.19 ± 0.07	2.07 ± 1.01	2.12 ± 1.00	0.461	0.801	0.876
12 months						
MH plate C	0.38 ± 0.20	0.41 ± 0.19	0.39 ± 0.17	0.617	0.198	0.722
DH plate C	2.41 ± 1.10	2.18 ± 0.17	2.31 ± 1.08	0.420	0.809	0.666
24 months						
MH plate C	0.57 ± 0.17	0.49 ± 0.07	0.51 ± 0.11	0.077	0.262	0.538
DH plate C	2.59 ± 0.09	2.47 ± 0.71	2.61 ± 0.11	0.301	0.602	0.234

*MH plate C: Changes in ridge height at the mesial aspect; DH plate C: Changes in ridge height at the distal aspect.

## The state of soft tissue surrounding the implant

The PPD (mesial, buccal mid and distal), the incidence of SH, BOP (mesial, buccal mid, distal) in group A were all significant higher than group B. The PISm, PISd, PPD (distal), the incidence of SH, BOP (mesial, buccal mid, distal) in group A were all significant higher than group C. The PISm, PISd, PPD (mesial, buccal mid), the incidence of SH, BOP (mesial, distal) in group B were all significant higher than group C. The above data are presented in Table 5.

**Table 5.** The soft tissue condition around the implant after surgery

Index	A(n = 30)	B(n = 39)	C(n = 30)	*p*		
				A vs B	A vs C	B vs C
PISm (mean±sd)	1.9 ± 1.5	2.5 ± 2.0	1.1 ± 0.3	0.449	**0.014**	**<0.001**
PISd (mean±sd)	1.9 ± 1.5	2.1 ± 1.6	0.7 ± 0.5	0.967	**<0.001**	**<0.001**
PPD (mean±sd)						
Mesial-	2.73 ± 1.0	2.2 ± 0.4	2.8 ± 0.9	**0.026**	0.999	**0.009**
Buccal mid-	2.7 ± 1.0	1.5 ± 0.5	2.5 ± 0.5	**<0.001**	0.584	**<0.001**
Distal-	3.3 ± 1.0	2.0 ± 1.0	2.0 ± 0.3	**<0.001**	**<0.001**	0.999
SH[n(%)]	0(0)	27(69.2)	12(40.0)	**0.008**	**0.023**	**0.027**
BOP[n(%)]						
Mesial-	27(90.0)	8(20.5)	18(60.0)	**<0.001**	**0.012**	**0.002**
Buccal mid-	11(36.7)	0(0)	0(0)	**0.007**	**0.019**	0.787
Distal-	28(93.3)	8(20.5)	1(3.3)	**0.008**	**0.023**	**0.027**

*PISm: Mesial papilla index; PISd: Distal papilla index; PPD: pocket probing depth; SH: Scar hyperplasia; BOP: Bleeding of probing.

## Patient satisfaction

The scores of chewing function, mucosal health, convenience of cleaning, voice function, comfort, robustness, overall satisfaction have no significant difference in group A VS group B, group B VS group C, group A VS group C. The scores of esthetic feeling have no significant difference in group A VS group B, group B VS group C. The score of esthetic feeling in group A was significantly higher than group C (P < 0.05). The above data are presented in Table 6.

**Table 6.** Subjective evaluation of patients after surgery

Index	A (n = 30)	B (n = 39)	C (n = 30)	*p*		
				A vs B	A vs C	B vs C
Chewing function						
Satisfaction rate[n(%)]	24(80.0)	34(87.2)	25(83.3)	0.435	0.739	0.659
Score(mean±sd)	3.2 ± 0.8	3.5 ± 0.8	3.5 ± 0.9	0.192	0.224	0.213
Esthetic feeling						
Satisfaction rate[n(%)]	27(90.0)	30(76.9)	20(66.7)	0.141	**0.035**	0.359
Score(mean±sd)	3.4 ± 0.7	3.2 ± 0.8	2.7 ± 1.0	0.178	0.218	0.222
Mucosal health						
Satisfaction rate[n(%)]	24(80.0)	29(74.4)	23(76.7)	0.577	0.754	0.824
Score(mean±sd)	4.1 ± 1.2	4.0 ± 1.3	3.9 ± 1.3	0.305	0.325	0.310
Convenience of cleaning						
Satisfaction rate[n(%)]	24(80.0)	31(79.5)	25(83.3)	0.958	0.739	0.682
Score(mean±sd)	3.3 ± 1.1	3.4 ± 1.0	3.2 ± 0.7	0.248	0.237	0.206
Voice function						
Satisfaction rate[n(%)]	25(83.3)	32(82.1)	26(86.7)	0.889	0.718	0.597
Score(mean±sd)	3.7 ± 1.1	3.6 ± 0.9	3.7 ± 1.0	0.240	0.264	0.228
Comfort						
Satisfaction rate[n(%)]	24(80.0)	32(82.1)	25(83.3)	0.830	0.739	0.889
Score(mean±sd)	3.6 ± 0.9	3.7 ± 1.0	3.6 ± 0.9	0.230	0.239	0.229
Robustness						
Satisfaction rate[n(%)]	28(93.3)	37(94.9)	27(90.0)	0.790	0.641	0.460
Score(mean±sd)	4.1 ± 1.0	4.2 ± 0.9	4.3 ± 1.0	0.241	0.264	0.248
Overall satisfaction						
Satisfaction rate[n(%)]	27(90.0)	35(89.7)	26(86.7)	0.972	0.688	0.698
Score(mean±sd)	25.4 ± 2.8	25.6 ± 3.0	25.0 ± 3.4	0.921	0.873	0.665

## Discussion

Adequate bone volume is the most important prerequisite for the long-term function and esthetic effect of dental implant restoration in the esthetic area of anterior teeth. However, due to traumatic, inflammatory and biological bone resorption, the alveolar bone lost in the horizontal or vertical direction after dental loss, making it difficult for the implant to obtain a good three-dimensional position in the dental area. Therefore, alveolar bone augmentation is particularly important in implant surgery. Currently, there are many methods for alveolar bone augmentation, including guided bone regeneration (GBR), onlay bone grafting, bone cleavage, etc. Among them, guided bone regeneration technology is the most widely used due to its relatively convenient operation and its ability to simultaneously guide osteogenesis in multiple directions [15–17].

Implant therapy has become one of the conventional methods for restoration of dentition defect or absence. The preservation or reconstruction of alveolar crest and gingival anatomical morphology is the prerequisite for satisfactory esthetic effect and long-term success of the restoration. Before the tooth extraction, the affected teeth with severe periodontitis and periodontal pulp lesions have different degrees of alveolar bone absorption. So, it is often necessary to adopt some surgical techniques for hard tissue increment. Then, the three-dimensional shape of alveolar crest was reconstructed, and the basic conditions for long-term stability and function of the implant (bone mass was abundant in the implant area) were also created. One of the important factors affecting the success of the implant operation is whether the implant can obtain good initial stability, which is indispensable to the amount of bone in the implant area. Preservation at the tooth extraction site refers to the implantation, support and filling of the alveolar bone with biomaterials immediately after tooth extraction. The purpose of preservation at the tooth extraction site includes blocking or slowing the absorption of alveolar bone, blocking the entry of gingival epithelial or fibrous tissue into the tooth extraction nest, guiding and promoting the bone regeneration of the tooth extraction nest, and achieving the preservation or increment of alveolar bone [18–20].

Causes of the lack of alveolar bone mass in patients with primary for patients with jaw bone and tooth body under the action of an external force damage, oral disease patients during treatment of tooth extraction, tooth to grow to repair time is too long, improper dental treatment, etc., these factors can lead to excessive occurred alveolar bone absorption and atrophy, and cause bone loss and is not enough to complete the planting area of tooth planting operation problem [21–24]. The commonly used methods of bone increment in oral implantation include bone extrusion, bone splicing, bone transplantation, membrane-guided bone tissue regeneration, maxillary sinus floor lifting, etc. In this study, a combination of Bio-OSS and Bio-GIDE was used to guide bone tissue regeneration. Bio-OSS bone powder is a deproteinized calf bone with similar porosity to human cancellous bone and good bone conductivity and inductivity. It is the most widely used bone substitute, and its osteogenic effect has been recognized. Bio-Gide membrane is a newly developed absorbable collagen membrane that does not need to be removed by secondary surgery. However, it lacks strength and is easy to collapse when implanted into bone defects alone. Bio-OSS is used to fill the bone defects to prevent the collapse of the membrane and provide a frame structure to guide the new bone tissue to grow inward [25–27].

In clinical practice, after guided bone regeneration through different surgical incisions, with the change of time, the curvature of the gingival surface on the lip side of the implant area and the shape fullness of alveolar bone changed to different degrees. CBCT examination also showed inconsistency in the residual amount of alveolar bone, gingival receding to different degrees, and scar hyperplasia. Therefore, we considered whether different incision designs had an effect on bone increment. In this study, we found that at the different time (immediately after surgery, 6 months, 12 months and 24 months): the width and height of labial bone at different implant margin (2 mm, 4 mm, 6 mm) has no significant difference in comparison of any two of the three groups (p > 0.05). Besides, we also concluded that the score of esthetic feeling in group A was significant higher than group C (P < 0.05); the soft tissue condition around the implant after surgery were better in group C than others two groups, group B better than group A.

The internal gingival crevicular incision (group C) was not designed with vertical relaxation incision, so there was no scar hyperplasia, and the gingival margin of the natural lip involved with the incision showed a small amount of different degrees of retreat. Small incisions (group A) refer to the medial gingival papilla incisions. The surgical area retained the gingival papilla of bilateral adjacent teeth in the absence of teeth, and there were vertical relaxation incisions on the medial gingival papilla. Therefore, after the wound healing, scars in the vertical incision area were relatively obvious, but the position of the adjacent gingival papilla remained almost unchanged. Large-scale incision (group B) refers to the internal gingival crevicular incision extending to 1–2 adjacent teeth outside the missing tooth area plus unilateral or bilateral vertical relaxation incision (angular incision or trapezoidal incision). Similarly, after the wound healing, scars in the vertical incision area are more obvious, and the stability of the position of the adjacent gingival nipple is not as good as small incision.

## Conclusion

The three groups have no significant difference on the influence bone increment. The soft tissue condition around the implant after surgery were better in internal gingival crevicular incision than others two incisions, large-scale incision better than small incisions.

## Highlights

1. Evaluating the influence of incision designs on bone increment of guided bone regeneration.
2. The three groups have no significant difference on the influence bone increment.

## References

[1] Bassi AP, Pioto R, Faverani LP, et al. Maxillary sinus lift without grafting, and simultaneous implant placement: a prospective clinical study with a 51-month follow-up. Int J Oral Maxillofac Surg. 2015;44(7):902–907. 25896082 10.1016/j.ijom.2015.03.016

[2] Beretta M, Poli PP, Grossi GB, et al. Long-term survival rate of implants placed in conjunction with 246 sinus floor elevation procedures: results of a 15-year retrospective study. J Dent. 2015;43(1):78–86. 25150106 10.1016/j.jdent.2014.08.006

[3] Burtscher D, Norer B, Dalla Torre D, et al. A 7-year prospective radiographic evaluation of marginal bone level around two different implant systems: a randomized clinical trial. Clin Oral Implants Res. 2015;26(11):1244–1249. 24995411 10.1111/clr.12444

[4] Silva LD, De Lima VN, Faverani LP, et al. Maxillary sinus lift surgery-with or without graft material? A systematic review. Int J Oral Maxillofac Surg. 2016;45(12):1570–1576. 27765427 10.1016/j.ijom.2016.09.023

[5] Avila-Ortiz G, Chambrone L, Vignoletti. F. Effect of alveolar ridge preservation interventions following tooth extraction: a systematic review and meta-analysis. J Clin Periodontol. 2019;46(21):195–223. 30623987 10.1111/jcpe.13057

[6] Danesh-Sani SA, Loomer PM, Wallace SS. A comprehensive clinical review of maxillary sinus floor elevation: anatomy, techniques, biomaterials and complications. Br J Oral Maxillofac Surg. 2016;54(7):724–730. 27235382 10.1016/j.bjoms.2016.05.008

[7] Sverzut AT, Rodrigues DC, Lauria A, et al. Clinical, radiographic, and histological analyses of calcium phosphate cement as filling material in maxillary sinus lift surgery. Clin Oral Implants Res. 2015;26(6):633–638. 24502772 10.1111/clr.12346

[8] Xavier SP, Santos Tde S, Sehn FP, et al. Maxillary sinus grafting with fresh frozen allograft versus bovine bone mineral: a tomographic and histological study. J Craniomaxillofac Surg. 2016;44(6):708–714. 27107475 10.1016/j.jcms.2016.03.005

[9] Pisoni L, Lucchi A, Persia M, et al. Sinus lift: 3 years follow up comparing autogenous bone block versus autogenous particulated grafts. J Dent Sci. 2016;11(3):231–237. 30894978 10.1016/j.jds.2015.10.007 PMC6395278

[10] Tang DYY, Yew GY, Koyande AK, et al. Green technology for the industrial production of biofuels and bioproducts from microalgae: a review. Environ Chem Lett. 2020;18(6):1967–1985.

[11] Yew GY, Tan X, Chew KW, et al. Thermal-Fenton mechanism with sonoprocessing for rapid non-catalytic transesterification of microalgal to biofuel production. Chem Eng J. 2021;408:127–264.

[12] Corbella S, Taschieri S, Del Fabbro M. Long-term outcomes for the treatment of atrophic posterior maxilla: a systematic review of literature. Clin Implant Dent Relat Res. 2015;17(1):120–132. 23656352 10.1111/cid.12077

[13] Jang JW, Chang HY, Pi SH, et al. Approach for maxillary sinus membrane elevation with <4 mm of residual bone height: a case report. Int J Dent. 2018;2018:1063459. 30050574 10.1155/2018/1063459 PMC6046141

[14] Kao SY, Lui MT, Cheng DH, et al. Lateral trap-door window approach with maxillary sinus membrane lifting for dental implant placement in atrophied edentulous alveolar ridge. J Chin Med Assoc. 2015;78(2):85–88. 25287252 10.1016/j.jcma.2014.05.016

[15] Khoo KS, Chew KW, Yew GY, et al. Recent advances in downstream processing of microalgae lipid recovery for biofuel production. Bioresour Technol. 2020;304:122–996. 10.1016/j.biortech.2020.122996 32115347

[16] Aprile P, Letourneur D, Simon‐Yarza T. Membranes for guided bone regeneration: a road from bench to bedside. Adv Healthc Mater. 2020;9:19. 10.1002/adhm.202000707 32864879

[17] De Moura NK, Martins EF, Oliveira RLMS, et al. Synergistic effect of adding bioglass and carbon nanotubes on poly (lactic acid) porous membranes for guided bone regeneration. Mater Sci Eng C. 2020;117:111–327. 10.1016/j.msec.2020.111327 32919681

[18] Aoki N, Baba J, Iwai T, et al. Window approach with micross mini bone scraper for sinus floor elevation. J Maxillofac Oral Surg. 2018;17(3):291–295. 30034146 10.1007/s12663-017-1039-2 PMC6028330

[19] Fermergård R, Åstrand P. Osteotome sinus floor elevation without bone grafts–a 3-year retrospective study with Astra Tech implants. Clin Implant Dent Relat Res. 2012;14(2):198–205. 19906268 10.1111/j.1708-8208.2009.00254.x

[20] Recent MA-D. Trends in sinus lift surgery and their clinical implications. Clin Implant Dent Relat Res. 2016;18(1):204–212. 25274014 10.1111/cid.12275

[21] Cardoso CL, Curra C, Santos PL, et al. Current considerations on bone substitutes in maxillary sinus lifting. Revista Clínica de Periodoncia. 2016;9(2):102–107.

[22] Corbella S, Taschieri S, Weinstein R, et al. Histomorphometric outcomes after lateral sinus floor elevation procedure: a systematic review of the literature and meta-analysis. Clin Oral Implants Res. 2016;27(9):1106–1122. 26452326 10.1111/clr.12702

[23] Danesh-Sani SA, Engebretson SP, Mn J. Histomorphometric results of different grafting materials and effect of healing time on bone maturation after sinus floor augmentation: a systematic review and meta-analysis. J Periodontal Res. 2017;52(3):301–312. 27534916 10.1111/jre.12402

[24] Si MS, Shou YW, Shi YT, et al. Long-term outcomes of osteotome sinus floor elevation without bone grafts: a clinical retrospective study of 4-9 years. Clin Oral Implants Res. 2016;27(11):1392–1400. 26754021 10.1111/clr.12752

[25] De Molon RS, De Paula WN, Spin-Neto R, et al. Correlation of fractal dimension with histomorphometry in maxillary sinus lifting using autogenous bone graft. Braz Dent J. 2015;26(1):11–18. 25672378 10.1590/0103-6440201300290

[26] Kim YK, Lee J, Yun JY, et al. Comparison of autogenous tooth bone graft and synthetic bone graft materials used for bone resorption around implants after crestal approach sinus lifting: a retrospective study. J Periodontal Implant Sci. 2014;44(5). DOI:10.5051/jpis.2014.44.5.216 PMC4216397 25368809

[27] La Monaca G, Iezzi G, Cristalli MP, et al. Histomorphometric results of six biomaterials used in two-stage maxillary sinus augmentation model after 6-month healing. Biomed Res Int. 2018;2018:9430989. 30050947 10.1155/2018/9430989 PMC6040296

